# Osteoporosis Epidemiology Among Adults With Cerebral Palsy: Findings From Private and Public Administrative Claims Data

**DOI:** 10.1002/jbm4.10231

**Published:** 2019-10-07

**Authors:** Zachary P French, Michelle S Caird, Daniel G Whitney

**Affiliations:** ^1^ Department of Physical Medicine and Rehabilitation, Michigan Medicine University of Michigan Ann Arbor MI USA; ^2^ Department of Orthopedic Surgery, Michigan Medicine University of Michigan Ann Arbor MI USA; ^3^ Institute for Healthcare Policy and Innovation University of Michigan Ann Arbor MI USA

**Keywords:** CEREBRAL PALSY, EPIDEMIOLOGY, OSTEOPOROSIS

## Abstract

Individuals with cerebral palsy (CP) have an increased risk for the early development of osteoporosis; however, little is known about the epidemiology of osteoporosis for adults with CP, which is vital to inform clinical practice for osteoporosis prevention, treatment, and management. The purpose of this cross‐sectional study was to determine sex‐stratified prevalence of osteoporosis among adults with CP, as compared with adults without CP. Data from 2016 were extracted from Optum Clinformatics Data Mart (private insurance administrative claims data) and a random 20% sample from the fee‐for‐service Medicare (public insurance administrative claims data). Diagnostic codes were used to identify CP and osteoporosis diagnoses. Sex‐stratified prevalence of osteoporosis was compared between adults with and without CP for the following age groups: 18 to 30, 31 to 40, 41 to 50, 51 to 60, 61 to 70, and >70 years of age. The overall prevalence of osteoporosis was 4.8% for adults without CP (*n* = 8.7 million), 8.4% for privately insured adults with CP (*n* = 7,348), and 14.3% for publicly insured adults with CP (*n* = 21,907). Women and men with CP had a higher prevalence of osteoporosis compared with women and men without CP for all age groups. Finally, publicly insured women and men with CP had a higher prevalence of osteoporosis compared with privately insured women and men with CP for all age groups, except for the similar prevalence among the 18‐ to 30‐year age group. These findings suggest that osteoporosis is more prevalent among adults with CP compared with adults without CP. Study findings highlight the need for earlier screening and preventive medical services for osteoporosis management among adults with CP. © 2019 The Authors. *JBMR Plus* published by Wiley Periodicals, Inc. on behalf of American Society for Bone and Mineral Research

## Introduction

In the United States, an estimated 10.3% of adults over the age of 50 years have osteoporosis.^1^ Osteoporosis is a high‐burden medical condition characterized by low bone mass or poor bone quality. A major consequence of osteoporosis is increased risk for fragility fracture. Among older adults, fracture care represents a significant economic burden, accounting for 68% of the total cost of osteoporosis treatment.^2^, ^3^ Fracture is also a major cause of functional limitations,^4^ morbidity (eg, noncommunicable diseases),^5^ poor quality of life,^6^ and early mortality.^4^, ^7^, ^8^ Microsimulation forecasting models have estimated that improving osteoporosis identification by 20% among elderly women (an at‐risk population for osteoporosis) could prevent 2.6 million fractures from 2018 to 2040 (assuming adequate treatment would be applied), which could lead to reducing cumulative osteoporosis‐related costs of nearly $42 billion over the same time period.^9^ Although osteoporosis and osteoporotic fractures are more commonly studied among postmenopausal women and adults over the age of 65 years, other segments of the population are vulnerable to fracture and warrant attention.^10^ For example, the risk for developing osteoporosis is higher for populations that have pediatric‐onset physical disabilities, such as cerebral palsy (CP).^11^, ^12^

CP is the most common physical disability in children affecting approximately 3.3 per 1000 children in the United States.^13^, ^14^, ^15^ CP follows from an insult or malformation of the developing central nervous system near the time of birth,^16^ leading to chronic and altered muscle tone,^17^ muscle contractures,^18^ dystonia,^19^ and low levels of physical activity.^20^ During development, low mechanical loading precipitates skeletal adaptation among children with CP, including an underdeveloped trabecular bone microarchitecture,^21^ thin cortices,^22^ and low bone strength.^20^ Despite knowledge of skeletal pathology and low peak bone mass attainment throughout growth and development, very little is known about the pathogenesis of skeletal fragility and the clinical care needed to prevent and manage osteoporosis and osteoporotic fractures among adults with CP.

The prevalence of osteoporosis in adults with CP has been recently reported to be 8.0%, 10.3%, 14.5%, and 25.9% for adults aged 18 to 30, 31 to 40, 41 to 50, and >50 years, respectively.^23^ However, inferences are limited as these data came from a single medical center in southeast Michigan, were not stratified by sex, and did not have controls to determine the extent of the osteoporosis‐related disparity. Moreover, risk of fracture is more than two times higher among privately insured young and middle‐aged adults (18 to 64 years) with CP compared with adults without CP.^24^ Taken together, there is a critical need to characterize the epidemiology of osteoporosis among individuals with CP throughout the adult lifespan, which can assist clinical care and public health surveillance for this underserved population. For example, knowing this information can help to identify the age at which osteoporosis is occurring, which can assist treatment strategies for the prevention of osteoporosis and management of osteoporosis and its sequela (eg, fracture) for this vulnerable population. Accordingly, the purpose of this study was to determine sex‐stratified prevalence of osteoporosis among adults with CP, as compared with adults without CP, using nationwide private and public administrative claims data. We hypothesized that women and men with CP would have a higher prevalence of osteoporosis compared with women and men without CP across the adult lifespan.

## Materials and Methods

### Data sources

Data for this study were extracted from private and public administrative claims data from the year 2016. Optum Clinformatics Data Mart Database (OptumInsight, Eden Prairie, MN, USA) provided deidentified information for privately insured beneficiaries. A random 20% sample of the Medicare fee‐for‐service database from the Centers for Medicare & Medicaid Services provided deidentified information for publicly insured beneficiaries. Because the data are deidentified, the local institutional review board approved this study as nonregulated.

### Sample selection

Beneficiaries who were 18 years of age or older, had 12 full months of continuous enrollment in at least one health plan, and had at least one medical service utilization in 2016 were initially included for analysis. We excluded Medicare beneficiaries covered by HMO plans because of incomplete claims, which could bias prevalence estimates. Beneficiaries who had unknown or missing data for sex were excluded (*n* = 991 from Optum, <0.01% of total sample).

International Classification of Diseases, Tenth Revision, Clinical Modification (ICD‐10) codes were used to identify all medical conditions, which are used for reimbursement purposes. ICD‐10 codes are entered into the billing system by healthcare providers. Information regarding how diagnoses were made is not available in administrative claims data. CP was identified by at least one medical claim using the G80 family of ICD‐10 codes (seven codes), covering all diagnostic subtypes of CP (eg, spastic quadriplegic, tetraplegic). Data regarding severity of CP using common clinical measures (eg, gross motor function classification system) are not available in administrative claims. Further, more than 70% of the cohort had “other” or “unspecified” CP, thus not allowing us to stratify or account for the clinical subtypes of CP (eg, spasticity/athetoid, hemiplegic) in the current study. However, data from Optum likely reflects the higher‐functioning segment of the CP population (eg, mild to moderate forms of CP).^24^ Therefore, insurance coverage will be used to stratify results. Using a single medical claim to identify a pediatric‐onset disability using administrative claims data has shown approximately 80% positive predictive value and 99% sensitivity.^25^

Beneficiaries without any medical claims for CP represented the group without CP (ie, control subjects), and were extracted from the Optum data source only. Using Optum to extract claims for the group without CP was performed to enhance the representativeness of our sample of adults without CP, as enrollment criteria for Medicare among individuals under 65 years of age requires permanent disability, such as end‐stage renal disease.

### Osteoporosis

A medical diagnosis of osteoporosis was identified by using at least one claim (ICD‐10 codes) for (1) osteoporosis without current pathological fracture (M81 family; 3 codes), or (2) osteoporosis with current pathological fracture (M80 family; 276 codes). Validation of identifying beneficiaries with osteoporosis from administrative claims data using diagnostic codes has been reported. Leslie and colleagues^26^ found that a case definition of at least one claim for osteoporosis had approximately 70% sensitivity, 95% specificity, and 92% positive predictive value for a one‐year period, which was better or similar to other case definitions developed by experts in the field.

### Sociodemographic and socioeconomic variables

Sociodemographic and socioeconomic variables that were available and reported in the same manner from both data sources included age and sex. Other confounding variables were not considered for covariate adjustment to limit bias for reasons such as they had not been reported in both data sources (eg, education level), they had not been reported in the same manner (eg, race), or they had missing data on over 20% of the cohort (eg, income).

### Statistical analysis

Descriptive characteristics were summarized using mean (SD) for continuous variables and percentage for categorical variables. Group differences (ie, privately insured adults with CP, publicly insured adults with CP, adults without CP) in descriptive characteristics and unadjusted prevalence of osteoporosis were examined using independent *t* tests or chi‐square tests.

We performed direct age‐standardization^27^ for osteoporosis for each group. The 2016 US adult population was used as a standard population. The U.S. Census Bureau released a table on age (5‐year age brackets) and sex composition in the US for 2016.^28^ To make use of the population table in 5‐year age groups, it was assumed that age was evenly distributed within the 15‐ to 19‐year age bracket. Therefore, because 6.8% of US males were 15 to 19 years old, it was assumed that 2.72% males were 18 to 19 years old [6.8% × (2/5)]. A similar approach was performed for females.

To examine the prevalence of osteoporosis across age and sex, age was stratified into the following categories to represent different stages of the adult lifespan, as previously described for adults with CP^23^, ^29^ and the general population^2^: 18 to 30, 31 to 40, 41 to 50, 51 to 60, 61 to 70, and >70 years of age.

To determine the statistical significance for this large sample, *p* ≤ 0.005 (two‐tailed) was used as recommended by a coalition of methodologists to detect new discoveries.^30^, ^31^ All analyses were performed using SAS version 9.4 (SAS Institute, Cary, NC, USA).

## Results

The final sample consisted of 8,732,455 privately insured adults without CP, 7,348 privately insured adults with CP, and 21,907 publicly insured adults with CP. Descriptive characteristics for study participants are presented in Table 1. Both privately and publicly insured adults with CP had a younger age and a lower proportion of females compared with adults without CP. The unadjusted prevalence of osteoporosis was significantly higher for publicly insured adults with CP (14.3%) compared with privately insured adults with CP (8.4%; *p* < 0.005) and adults without CP (4.8%; *p* < 0.005). The unadjusted prevalence of osteoporosis was significantly higher for privately insured adults with CP compared with adults without CP (*p* < 0.005). For women, the age‐standardized prevalence of osteoporosis was 4.9% for individuals without CP, 10.4% for privately insured individuals with CP, and 16.5% publicly insured individuals with CP. For men, it was 0.6%, 4.7%, and 9.0%, respectively.

**Table 1:** Descriptive Characteristics of Study Participants With and Without Cerebral Palsy (CP)

	Without CP	With CP	With CP
	Private insurance (*n* = 8,732,455)	Private insurance (*n* = 7,348)	Public insurance (*n* = 21,907)
	Point estimate	Point estimate	Point estimate
Age, mean (SD)	55.2 (18.6)	49.8 (18.2)*	51.2 (15.6)* ^,^ **
18–30 years, %	12.4	20.0	10.8
31–40 years, %	13.1	14.1	17.2
41–50 years, %	14.5	14.5	20.1
51–60 years, %	17.0	19.6	22.9
61–70 years, %	18.9	18.5	17.7
>70 years, %	24.1	13.3	11.3
Sex, %			
Female	55.3	49.2*	47.7*
Male	44.7	50.8*	52. 3*
Osteoporosis, %	4.8	8.4*	14.3* ^,^ **

* Different compared with adults without CP, *p* < 0.005.

** Different compared with privately insured adults with CP, *p* < 0.005.

**Figure 1:**
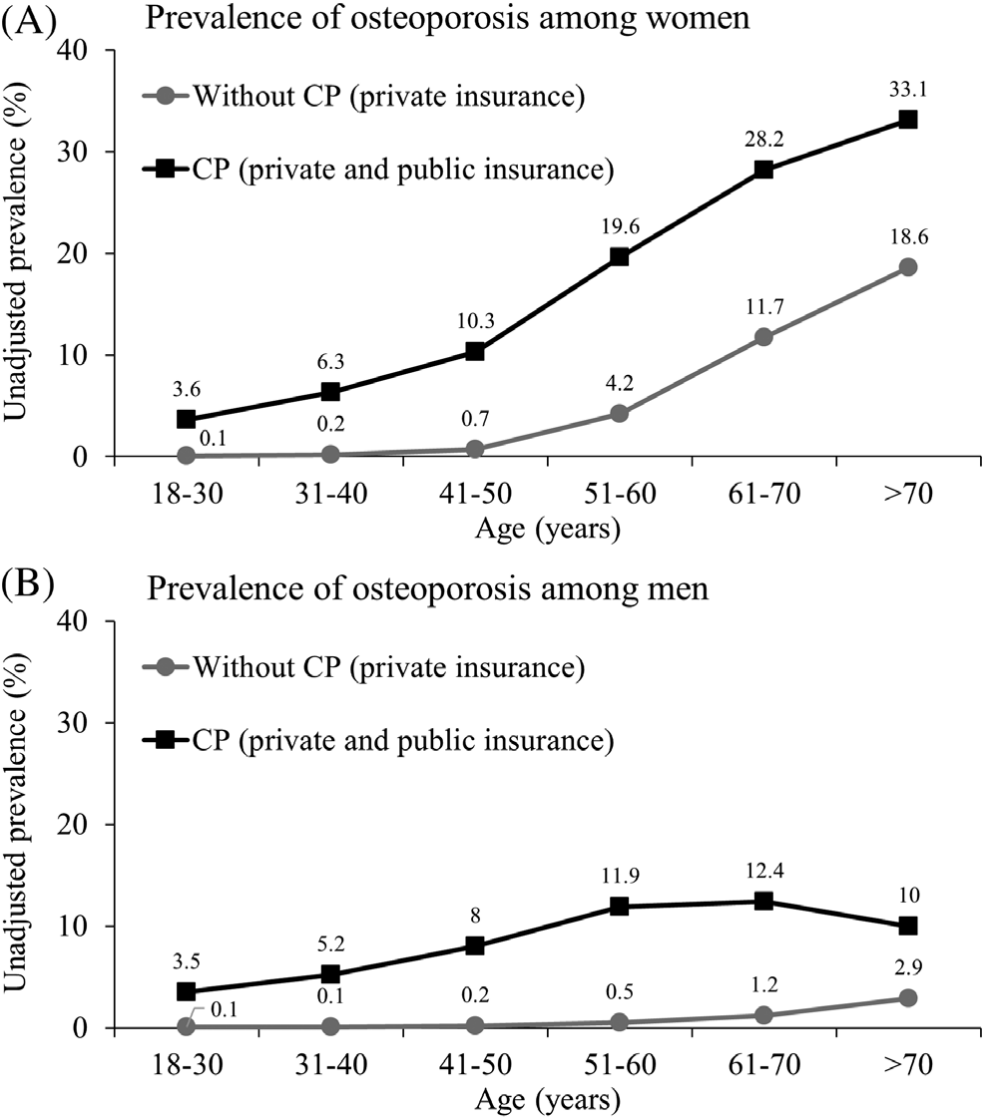
Prevalence of osteoporosis among women (*A*) and men (*B*) with and without cerebral palsy (CP).

Unadjusted prevalence of osteoporosis across age strata is presented in Fig. 1 A for women and Fig. 1B for men. There was an increasing trend of osteoporosis for adults without CP and for the combined sample of adults with CP. Women and men with CP had a higher prevalence of osteoporosis compared with women and men without CP for all age groups. For women without CP, the prevalence of osteoporosis was 0.1% for 18 to 30 years and 18.6% for >70 years. For women with CP, the prevalence of osteoporosis was 3.6% for 18 to 30 years and 33.1% for >70 years. For men without CP, the prevalence of osteoporosis was 0.1% for 18 to 30 years and 2.9% for >70 years. For men with CP, the prevalence of osteoporosis was 3.5% for 18 to 30 years and 10.0% for >70 years.

**Figure 2:**
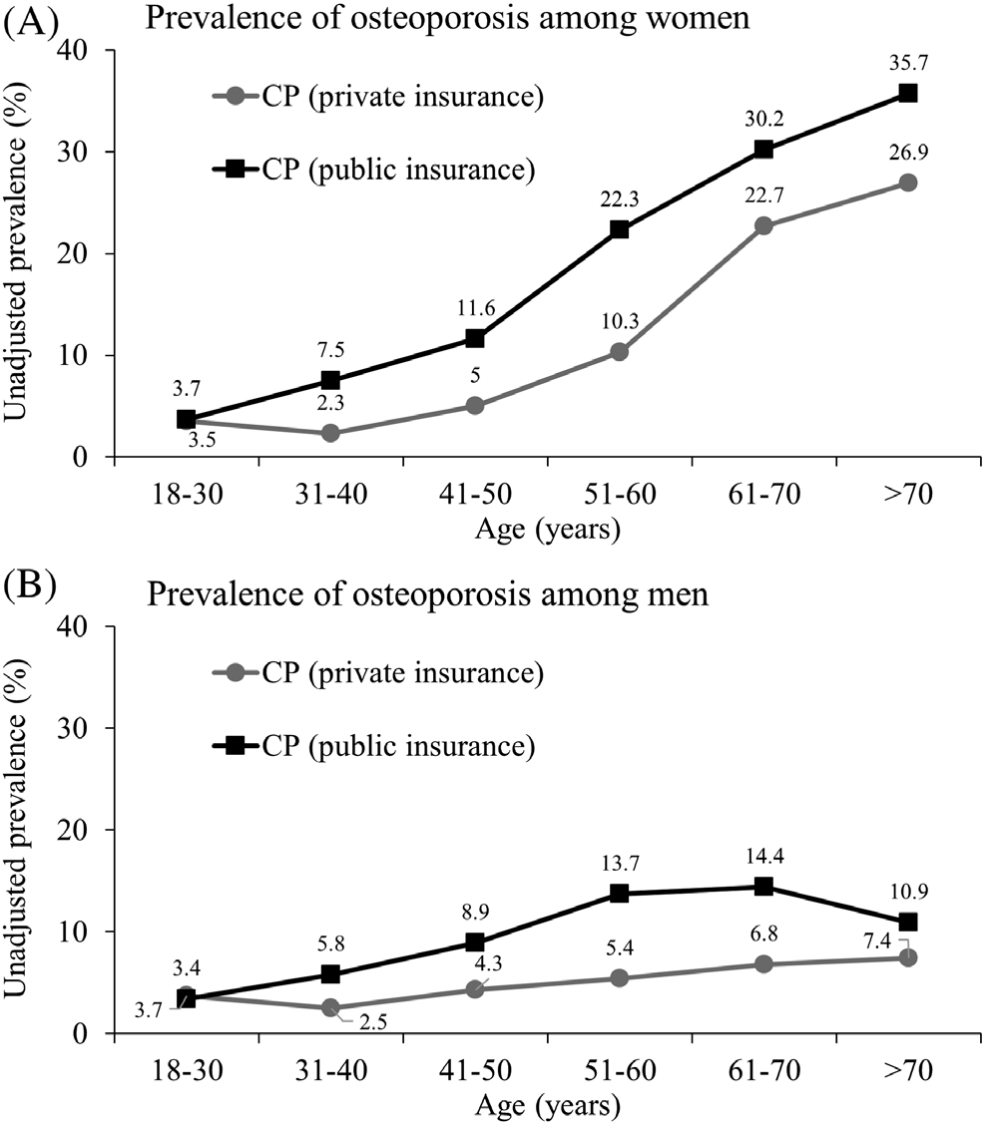
Prevalence of osteoporosis among women (*A*) and men (*B*) with cerebral palsy (CP) by insurance coverage.

The unadjusted prevalence of osteoporosis across age strata for adults with CP that had private and public insurance is presented in Fig. 2 A for women and Fig. 2B for men. Both groups with CP had a higher prevalence of osteoporosis compared with adults without CP. Publicly insured women and men with CP had a higher prevalence of osteoporosis compared with privately insured women and men with CP for all age groups, except for the 18‐ to 30‐year age group.

## Discussion

The chief finding of this study was that women and men with CP had a higher prevalence of osteoporosis compared with women and men without CP. Although the prevalence of osteoporosis was higher for publicly and privately insured adults with CP compared with adults without CP for each age group and for both sexes, the prevalence was more pronounced among individuals that had public insurance. These findings are important because the updated 2018 evidence‐based guidelines set forth by the U.S. Preventive Services Task Force^32^ recommends screening for osteoporosis in all adults 65 years of age and older, but provides no recommendations for the growing adult CP population^33^, ^34^ or other adult populations with pediatric‐onset disabilities and resultant skeletal fragility. Our study findings provide large, national‐level data to support the need for: (1) earlier preventive and health management services for osteoporosis; (2) future research to investigate osteoporosis‐related burdens specific to the adult population with CP; and (3) clinical studies to maximize musculoskeletal development throughout growth for children with CP to offset the early development of osteoporosis.

The prevalence of osteoporosis among women and men without CP from the current study is slightly lower or similar to that previously reported for adults 50 years of age and older from the National Health and Nutrition Examination Survey (NHANES) 2005 to 2010 data.^1^ The reason our data showed a slightly lower prevalence for some age groups is that we used insurance‐based claims for a medical diagnosis of osteoporosis (criteria for diagnosis are not stated in claims data), whereas NHANES osteoporosis was identified through DXA, thus better detecting osteoporosis. It is not uncommon to underdetect osteoporosis in the clinical setting, which is where administrative claims data are derived. Nevertheless, our sample of adults without CP included 8.7 million beneficiaries of private insurance in 2016: a sample that likely reflects the general employed population without severe medical conditions that require frequent healthcare utilization.

Data from the current study represent the largest known sample of claims data for adults with CP evaluated for osteoporosis prevalence. We found that the prevalence of osteoporosis was higher among adults with CP than adults without CP, and the prevalence increased with age, except for men >70 years old with public insurance. The reason for the dip in osteoporosis prevalence among men >70 years old with public insurance may be because of a “survivor” effect. Although both privately and publically insured adults with CP had lower prevalence at the oldest age group, the publically insured sample had even lower prevalence compared with the privately insured sample. Adults with CP have lower life expectancy,^35^ and those covered by Medicare are presumably less healthy than those covered by Optum, which is caused by a variety of factors including medical need and health plan‐specific enrollment criteria. Therefore, the men with CP >70 years with public insurance may be abnormally healthier than what would be expected for a CP diagnosis at that age. Nevertheless, our prevalence trends are concordant with previous research in southeast Michigan showing that young adults (18 to 30 years) with CP have a musculoskeletal morbidity profile that is 10 times higher than young adults without CP,^29^ with the trend of musculoskeletal morbidity becoming even more prevalent with older age.^23^

Our findings reflect long‐term consequences of osteoporosis development by numerous factors that are inherent and a resulting sequela of a CP diagnosis. Premature birth,^36^ poor oromotor function,^37^ inadequate nutrition and calcium intake,^38^ anticonvulsant use,^39^ and nonambulation or immobility are commonly seen among individuals with CP, and are associated with low bone mineral density. Moreover, underdeveloped skeletal muscle^20^, ^40^ can lead to low mechanical loading during development,^41^ exacerbating the inadequate accrual of bone mineral and structure.^21^ With aging, factors contributing to osteoporosis only get worse. Individuals with CP experience reduced ambulatory ability^42^ and develop other noncommunicable diseases^29^ that contribute to early development of osteoporosis. Moreover, adults with CP are susceptible to complications associated with osteoporosis, including increased fracture risk, which is evident even after accounting for osteoporosis.^24^

Study findings highlight the need for osteoporosis surveillance for adults with CP. DXA is the gold‐standard osteoporosis screening methodology and is currently only recommended for adults aged 65 and over, or younger women with certain risk factors (smoking, low body mass index, daily use of alcohol).^32^ DXA has been shown to be a technically feasible test for individuals with CP, despite often relying on the imaging of anatomical sites that are commonly sites of previous surgery in this population.^43^ Earlier screening for skeletal health may allow for earlier detection, preventive services, and rehabilitation efforts to prevent or attenuate the burden of osteoporosis, which is needed for adults with CP, although this notion is confounded by whether available osteoporosis‐related treatment strategies actually work in reducing skeletal fragility for adults with CP. More research on this topic is warranted.

The major strength of this study was that we extracted data from both private (Optum) and public (Medicare) administrative claims. In doing so, we ascertained a very large nationwide sample of adults with CP, which not only increases the external validity of our study findings, but also provides robust prevalence estimates.

However, this study also had several limitations. First, administrative claims data can be subject to inaccurate coding that could affect interpretation. Second, we used a single claim to define CP and osteoporosis. Previous validation studies have shown that two or more claims for a medical condition tend to improve accurate identification of that medical condition.^25^, ^44^ However, accurately identifying medical conditions depends on the number of years for the study period^26^ and the medical condition examined.^25^, ^26^, ^45^ A single claim‐based definition for identifying a pediatric‐onset disability and osteoporosis performs better compared with other medical conditions, with positive predictive values of approximately 80%^25^ and up to 92%,^26^ respectively. Third, we did not account for potential confounding factors, such as ethnicity, geographic region, or other socioeconomic status variables (eg, education level). Although this was not the purpose of the present work, future research is needed to identify if socioeconomic status plays a role in the development or worsening of osteoporosis among adults with CP. Fourth, we were unable to account for severity or type of CP as more than 70% of the cohort from Optum^46^ and Medicare (unpublished observations) had “other” or “unspecified” CP. In light of this limitation, we stratified results by insurance coverage to serve as a proxy for severity of CP, as private insurance likely reflects the higher‐functioning segment of the CP population and public insurance likely reflects the lower‐functioning segment of the CP population. This speculation is based on differences in enrollment criteria between insurance types, medical needs of individuals with CP based on insurance coverage, and prevalent chronic diseases for adults with pediatric‐onset disabilities (higher among publicly vs privately insured), including CP.^47^

In conclusion, adults with CP have a higher prevalence of osteoporosis throughout the adult lifespan compared with adults without CP. Further, publicly insured women and men with CP showed a higher prevalence of osteoporosis than privately insured women and men with CP. These data can inform future public health and clinical practice guidelines for screening and management of osteoporosis in patients with CP. Future research is needed to identify effective interventions to attenuate the burden of osteoporosis for individuals with CP.

## Disclosures

All authors have nothing to disclose.
